# Recurring priapism may be a symptom of voiding dysfunction – case report and literature review

**DOI:** 10.1590/S1677-5538.IBJU.2015.0435

**Published:** 2016

**Authors:** Lisieux Eyer de Jesus, Leonardo Teixeira, André Bertelli

**Affiliations:** 1 Departamento de Cirurgia Pediatrica - Hospital Universitário Antônio Pedro, Niterói, RJ, Brasil; 2 Departamento de Cirurgia Pediatrica Hospital Federal dos Servidores do Estado do Rio de Janeiro, RJ, Brasil; 3 Departamento de Urologia - Hospital Universitário Antônio Pedro, Niterói, RJ, Brasil

**Keywords:** Priapism, Child, Urinary Bladder, Overactive

## Abstract

Recurring priapism is rare in pre-pubertal children and may be attributed to multiple causes. We propose that voiding dysfunction (VD) may also justify this symptom and detail a clinical case of recurring stuttering priapism associated to overactive bladder that completely resolved after usage of anticholinergics and urotherapy. Sacral parasympathetic activity is responsible for detrusor contraction and for spontaneous erections and a relationship between erections and bladder status has been proved in healthy subjects (morning erections) and models of medullar trauma. High bladder pressures and/or volumes, voiding incoordination and posterior urethritis can potentially trigger reflex erections.

## INTRODUCTION

Recurring priapism is rare in pre-pubertal children and is most frequently caused by sickle cell disease (SCD)(65%), leukemia (10%), drugs (notably exogenous testosterone and psychiatric medications (5%) and penile trauma (10%) (1). A causal association between recurring priapism and voiding dysfunction (VD) has never been recognized.

We report a clinical case of recurring priapism secondary to VD, review the pertinent literature and pathophysiological mechanisms involved.

## MATERIAL AND METHODS

We report a case of stuttering priapism treated with oral anticholinergics and urotherapy (patient/family education, timed voiding and generous daily hydration). Literature data were retrieved from PUBMED, using the keywords priapism AND intermittent AND “voiding dysfunction” and are reviewed in the discussion session.

## CASE REPORT

A five years-old white male was referred to Urology Clinics to investigate recurring stuttering priapism, present for 2 months. The child reported painful erections during circa 5 min each, with simultaneous glans and corpora engorgement, three times a week, associated to hypogastric pain, consistent with stuttering priapism (2). Physical and neurological examination, growth and development landmarks were normal. As no episodes of priapism occurred in the presence of members of the health team the description of the problem depended on the mother´s report. External genitalia, vertebral column and lumbar examination were also normal. There was no history of penile or pelvic trauma. The mother denied previous or familiar anemia. There was a history of repetitive urinary tract infections (UTI) and intermittent urinary flow. The child was toilet trained for feces and urine at 1.5 years-old. His past history also showed asthma (treated with loratadin, inhaled fluticasone and inhaled salbutamol as needed), chronic gastritis and reflux esophagitis (treated with omeprazole). The family history revealed a sister presenting type 1 diabetes.

The patient presented no anemia. Hemoglobin electrophoresis excluded SCD (97% hemoglobin A, 3% hemoglobin A2). Serum creatinine and electrolytes were normal, as repetitive urine tests, except for the presence of mucus. Urinary culture was negative. Urinary calcium/creatinine, urinary protein/creatinine, proteinuria, calciuria were normal (respectively 0.03, 0.16, 0.19 mg/kg/h, 0.09 mg/kg/h). Abdominal and pelvic/perineal doppler ultrasound was normal, without significant post-voiding residuals. Cavernous blood gas analysis was not considered for differential diagnosis, as the episodes were short and intermittent and there was no evidence of an ischemic cause.

A month later the child still referred very frequent recurring stuttering painful priapism. A voiding diary showed abnormally high voiding frequency (> hourly), episodes of urge-incontinence and intermittent flow. The mother denied enuresis or constipation. The child refused uroflowmetry. According to International children´s continence society (ICCS) recommendations (2), we started him on oral oxybutinin (0.2 mg/kg bid) and urotherapy, with the clinical diagnosis of overactive bladder and possible detrusor-sphincter incoordination (the mother described intermittent flow).

Four months after starting oral anticholinergics the mother reported only one self-limited priapism episode. Daily voiding frequency was ameliorated (2/2 h) and urge-incontinence resolved, but the child developed nocturia. Six months after starting anticholinergics priapism and urinary complaints were clinically resolved.

## DISCUSSION

Childhood priapism is rare and typical of preadolescents/adolescents, presenting exceptionally in preschool children. The disease has multiple etiologies that vary among ages and populations. High flow priapism is mainly caused by penile/perineal trauma, cavernous vasoactive drugs injections, poisoning (scorpion, spider and snake venom), some drugs (notable psychoactive medications, anticoagulants, sildenafil and propofol), local infection (cavernositis), epidural anesthesia and medullary tumors. Low flow priapism is mainly caused by SCD, leukemia and hypercoagulable states. “Idiopathic priapism” remains the diagnosis in at least 10% of pediatric cases (2). In Brazil the large majority of priapism episodes in children are caused by SCD, due to the ethnical distribution of our population. Only a quarter present in pre-pubertal children (2). Treatment depends on the cause of the disease.

Repetitive stuttering priapism, as presented in our index case, is also typically secondary to SCD. Other causes remain ill-defined. Those cases are probably underreported due to the self-limited nature of the episodes.

A voiding history is not normally asked for in priapism cases, as the possible relationship between VD and priapism is not normally considered (3). Ironically, one of the first recommendations for patients to treat an episode of SCD priapism is to urinate, in order to induce detumescence (4). Also, the linkage between morning erections and full bladders, explained by sacral neural reflexes, are lay knowledge.

The parasympathetic neural system starts the erection mechanisms that depend on a normal balance of the autonomic nervous system. Similar neural mechanisms coordinate normal voiding, added by voluntary nerve responses and reflexes mediated by the pudendal nerve. A relationship between prolonged erection and perineal muscles´ spasms has been proved in animals (5).

The voiding defense reflex consists of a perineal contraction in the event of inappropriate detrusor activity and is repetitively triggered in overactive bladder cases in children, probably leading to detrusor-sphincter incoordination (DSI) in the long term. DSI may induce high pressures in the posterior urethra, high voiding pressures, incomplete bladder emptying and vasal urinary reflux with secondary epididymitis and urethritis (6–10). High bladder pressures or volumes and posterior urethritis can potentially trigger reflex erections, so linking VD and repetitive priapism.

We acknowledge that our diagnosis of VD is entirely based on clinical data, but this is a functional disease, and the recommendations of ICCS are not to use urodynamic evaluation for diagnosis, after excluding possible neurological or anatomical problems, unless for irresponsive children (3). The presentation of this clinical case should possibly be followed be a research project to prospectively investigate the frequency of reflex erections and priapism in Pediatric cases of VD, as compared to the normal population.

Recurring priapism in children may be secondary to VD. The diagnosis of VD should be excluded in cases of recurring priapism. Treatment of VD may resolve stuttering repetitive priapism.
